# Dopamine Modulates Insulin Release and Is Involved in the Survival of Rat Pancreatic Beta Cells

**DOI:** 10.1371/journal.pone.0123197

**Published:** 2015-04-17

**Authors:** Maria Jose Garcia Barrado, Maria Carmen Iglesias Osma, Enrique J. Blanco, Marta Carretero Hernández, Virginia Sánchez Robledo, Leonardo Catalano Iniesta, Sixto Carrero, Jose Carretero

**Affiliations:** 1 Department of Physiology and Pharmacology, Faculty of Medicine, University of Salamanca, Salamanca, Spain; 2 Department of Human Anatomy and Histology, Faculty of Medicine, University of Salamanca, Salamanca, Spain; 3 Laboratory of Neuroendocrinology, Institute of Neurosciences of Castilla y León, and Laboratory of Neuroendocrinology and Obesity of IBSAL, University of Salamanca, Salamanca, Spain; La Jolla Institute for Allergy and Immunology, UNITED STATES

## Abstract

The local synthesis of dopamine and its effects on insulin release have been described in isolated islets. Thus, it may be accepted that dopamine exerts an auto-paracrine regulation of insulin secretion from pancreatic beta cells. The aim of the present study is to analyze whether dopamine is a regulator of the proliferation and apoptosis of rat pancreatic beta cells after glucose-stimulated insulin secretion. Glucose stimulated pancreatic islets obtained from male Wistar rats were cultured with 1 or 10 μM dopamine from 1 to 12 h. Insulin secretion was analyzed by RIA. The cellular proliferation rate of pancreatic islets and beta cells was studied with immunocytochemical double labelling for both insulin and PCNA (proliferating cell nuclear antigen), and active caspase-3 was detected to evaluate apoptosis. The secretion of insulin from isolated islets was significantly inhibited (p<0.01), by treatment with 1 and 10 μM dopamine, with no differences between either dose as early as 1 h after treatment. The percentage of insulin-positive cells in the islets decreased significantly (p<0.01) after 1 h of treatment up to 12 h. The proliferation rate of insulin-positive cells in the islets decreased significantly (p<0.01) following treatment with dopamine. Apoptosis in pancreatic islets and beta cells was increased by treatment with 1 and 10 μM dopamine along 12 h. In conclusion, these results suggest that dopamine could modulate the proliferation and apoptosis of pancreatic beta cells and that dopamine may be involved in the maintenance of pancreatic islets.

## Introduction

Dopamine is a neurotransmitter that plays a critical role in neurological and psychiatric disorders [1] and it is involved in various physiological functions, including modulation of the endocrine system. Insulin secretion elicited by glucose metabolism can be modulated by parasympathetic and sympathetic neurotransmitters [2–4].

Treatment with the dopamine precursor L-dopa in patients with Parkinson’s disease reduces insulin secretion in oral glucose tolerance tests [5], but studies in humans do not suggest that diabetes would be a preceding risk factor for Parkinson’s disease [6]. In rodents, a single injection of L-dopa results in the accumulation of dopamine in beta cells and the inhibition of insulin secretory responses [7,8].

The literature contains conflicting reports about the effects of dopamine analogues on glucose-stimulated insulin release in isolated islets. Several authors consider that dopamine analogues would inhibit glucose-stimulated insulin release [9], whereas others have reported an enhancement of insulin secretion upon acute dopamine accumulation [3]. These controversies can be explained because different doses of dopamine can induce opposite effects on insulin secretion [10]. Moreover, several classical neurotransmitters that act directly on beta cells could function indirectly by enhancing the signals generated by the beta cell glucose-sensing apparatus [11].

In contrast, the non-selective and selective antagonism of receptors involved in islet dopamine signalling generally induces increased glucose-stimulated insulin secretion [12]. This suggests that beta cells might be responsive to dopamine directly. Additionally, dopamine inhibits glucose-stimulated insulin secretion without modifying intracellular cAMP levels and it decreases the levels of cytosolic calcium [13] and reduces the frequency of intracellular calcium fluctuations [14].

Because the presence in beta cells of the enzymes responsible for the synthesis, metabolization and storage of dopamine (TH, DOPA, MAO and VMAT-2) has been reported [15–18], it can be accepted that dopamine could be produced from beta cells and it would exert an auto-paracrine regulation of insulin secretion in these cells.

However, it has been speculated that the inhibition of glucose-stimulated insulin secretion induced by D2 agonist such as bromocriptine may occur through alpha2-adrenergic receptors [19]. Additionally, dopamine also act directly on dopamine receptors because the expression of D2, D3 and D4 dopaminergic receptors has been described in pancreatic islet cells [13,14,20–22].

The absence of dopaminergic inhibition in knockout d2^-/-^ mice induces a reduction in pancreatic beta cell mass, and decreased beta cell replication in 2-month-old mice has been reported [20], suggesting that the dopaminergic modulation of pancreatic beta cells can modulate the cellular proliferation and/or apoptosis of these cells. In the other tissues, has been demonstrated that the physiological effect of dopamine stimulation was different, dopaminergic activation significantly increased apoptosis in young, but not neonatal striatal neurons [23].

It is not clear if dopamine develops its effect on insulin secretion directly or modifying the population of pancreatic beta cells. The aim of the present study is determine whether dopamine is involved in the maintenance of beta pancreatic cells acting on the cellular proliferation and apoptosis of these cells. For these propose, effects of dopamine on rat isolated pancreatic islets after glucose-induced secretion of insulin were studied. With this in mind, different doses and time-points of dopaminergic inhibition were assayed.

## Materials and Methods

### Animals and treatments

Animal experimentation was performed according to the Guide for the Care and Use of Laboratory Animals of the National Institutes of Health (NIH Publication No. 85–23, revised 1996). All procedures were approved by the Committee for the Care and Use of Animals of the University of Salamanca which ensures compliance with national and European legislation regarding the use of animals in research (Spanish RD 53/2013 and 2010/63/EU). All animals were kept under standard stabling conditions (temperature 21±2°C, relative humidity 50±5%, controlled photoperiod of 14h light/10h darkness, food and water *ad libitum* with a balanced rat/mouse maintenance diet (Panlab).

The pancreatic islets were isolated as was described previously from our laboratory [24]. For these propose, Wistar male rats (Harlan Laboratories, Spain) of 8 months of age (175–200g body weight) were anesthetized by intraperitoneal injection of 25mg/kg pentobarbital (Sigma-Aldrich). After the animals were sacrificed, the pancreas was inflated by injecting 5 ml of medium containing 1.5 mg/ml of collagenase (Serva, Inmunogenetics, Spain) through the common pancreatic duct. Islet isolation was performed using sterile-filtered Krebs solution. After collagenase digestion, the islets were then microdissected and handpicked under a stereomicroscope to ensure high purity of the islet preparation. The islets were maintained on ice during the entire isolation except for 12 min of collagenase digestion. The sterile-filtered Krebs solution containing: 120mM NaCl, 4.8mM KCl, 2.5mM CaCl_2_, 1.2mM MgCl_2_, 24mM NaHCO_3_, 10mM glucose, 5mM HEPES. It was gassed with O_2_/CO_2_ (94/6) to maintain a pH of 7.4, and contained: 1 mg/ml BSA. To avoid contamination, 100 IU/ml penicillin, 100 μg/ml streptomycin and 0.3 μg/ml fungizone were added. The islets were then cultured overnight in 2.5 ml of RPMI 1640 medium (Life Technologies S.A.) containing 10mM glucose, 10% heat-inactivated FCS, 2 mmol/l glutamine, 100 IU/ml penicillin, and 100 μg/ml streptomycin [24].

### Insulin secretion

After overnight culture, treatment with or without dopamine was applied for different periods of time (1, 3, 6 and 12 hours) in batches of ten islets and these were maintained in RPMI 1640 medium, 10 mM glucose, 100 IU/ml penicillin, and 100 μg/ml streptomycin, and 1 or 10 μM of dopamine, and without FCS. At the end of culture, an aliquot of the medium was collected to measure the insulin concentration.

### Insulin content

The islets were recovered from the Petri dishes, and their insulin content was determined after extraction in acid-ethanol. Insulin was measured by rat double-antibody RIA (LINCO Research, Inc. USA). All experiments were carried out at 37°C.

### Immunocytochemical study

In one series of experiments, batches of fifty islets from adult rats were treated in the same conditions as those used for the analysis of insulin secretion. At the end of the treatment the islets were fixed by immersion in 4% paraformaldehyde in phosphate buffer (0.1 M, pH 7.4). Subsequently, the islets were counterstained by immersion in Mayer’s haematoxylin for macroscopic visualization, after which they were embedded in paraffin and 5-μm serial sections were obtained.

### Determination of cellular proliferation by double immunocytochemical labelling for insulin and Proliferating Cell Nuclear Antigen (PCNA)

The proliferation rate of insulin cells was determined by double immunocytochemical labelling for PCNA and insulin.

To determine PCNA-positive cells, the biotinylated-streptavidin-peroxidase immunocytochemical method was implemented in a similar way to what is described in [25]. Prior to developing the reaction, endogenous peroxidase was blocked with 0.05% H_2_0_2_ in methanol and non-specific reactions of the secondary antibody were blocked by incubation in normal goat serum (Dako, diluted 1:30). Sections were incubated overnight at 4°C with the mouse PC10 (PCNA) mAb (Dako, diluted 1:2000 in TBS, Trizma base, Sigma, 0.05M, pH 7.4, plus 0.09% NaCl). Biotinylated goat anti-mouse IgG (Dako, diluted 1:100, in TBS) and streptavidin-horseradish peroxidase complex (Dako, diluted 1: 100 in TBS) were applied successively at room temperature for 40 min respectively. The reactions were developed in freshly prepared 3–3’diaminobenzidine (3–3'DAB, Sigma) (0.025% in TRIS buffer containing 0.03% of H_2_0_2_). Because antibodies against PCNA and insulin were both monoclonal antibodies obtained from mice before the determination of insulin-positive cells, immunoglobulins were eluted by immersion in glycine buffer (0.1M, pH 2.2) at 4°C overnight. To determine insulin-positive cells, an immunofluorescence study was carried out. For this, sections were incubated with monoclonal mouse anti-insulin (diluted 1:1000 in TBS, Sigma) overnight at 4°C, followed by incubation with anti-mouse IgG CY3 conjugate developed in sheep (1:100 in TBS, Sigma) for 60 minutes at room temperature. Slides were mounted with fluoromount aqueous mounting medium (Sigma). Controls included substitution of the primary antibody by normal mouse serum or TBS, as well as omission of the secondary antibody; after both tests, no immunoreactivity was detected.

### Determination of cellular apoptosis by double immunocytochemical labelling for insulin and active Caspase-3

The apoptosis rate of insulin cells was determined by double immunocytochemical labelling for active Caspase-3 and insulin.

To determine insulin-positive cells, the biotinylated-streptavidin-peroxidase immunocytochemical method was carried out. For this, sections were incubated with monoclonal mouse anti-insulin (diluted 1:1000 in TBS, Sigma) overnight at 4°C, followed by incubation with biotinylated goat anti-mouse IgG (Dako, diluted 1:100, in TBS) and streptavidin-horseradish peroxidase complex (Dako, diluted 1: 100 in TBS), applied successively at room temperature for 40 min each. The reactions were developed in freshly prepared 3–3'DAB (0.025% in TRIS buffer containing 0.03% of H_2_0_2_). The second reaction for active caspase-3 was visualized by immunofluorescence. The sections were incubated with polyclonal rabbit anti-active caspase-3 (Sigma, diluted 1: 500 in TBS) overnight at 4°C, followed by incubation with biotinylated goat anti-rabbit IgG (Dako, diluted 1:100, in TBS) and Streptavidin-Cy3 complex (Sigma, diluted 1: 100 in TBS) applied successively at room temperature for 40 and 60 min, respectively.

### Morphometric analyses

The morphometric analysis of pancreatic insulin-positive cells was carried out on high-definition micrographs obtained from structurally well conserved cells at a final magnification x350, using Image J, NIH software. Only cells with intact cellular and nuclear contours in the section plane were selected.

In each section, the cellular and nuclear profiles of insulin-positive cells were plotted and measured in order to calculate the size of the cellular and nuclear areas of 1000 insulin-positive cells (100 cells per islet, from 10 islets per study group). The cells were selected at random and individually using the double-blind method. The values obtained are expressed in square microns.

### Quantification of insulin-positive and insulin and PCNA-positive cells

For the planimetric study, digital high-resolution micrographs at x140 final magnification of each islet analyzed were used (10 per treatment group). For each experiment and staining method, 4000 cells were counted randomly, and positivity for insulin and PCNA, either alone or together, was measured. Then, the percentages of positive cells were calculated. Quantification was carried out by the double-blind method. Similar procedure was employed to determine the percentage of apoptotic and non-apoptotic insulin-positive cells using active caspase-3.

### Statistical analyses

The results obtained were processed statistically using GraphPad Prism 5.0 and are expressed as arithmetic means ± standard errors of the means. Statistical significance was assessed with Student's t-test or one-way analysis of variance (ANOVA), corrected by the Newman–Keuls test. The results were considered significant for p < 0.05.

## Results

### Insulin secretion

**Fig 1:**
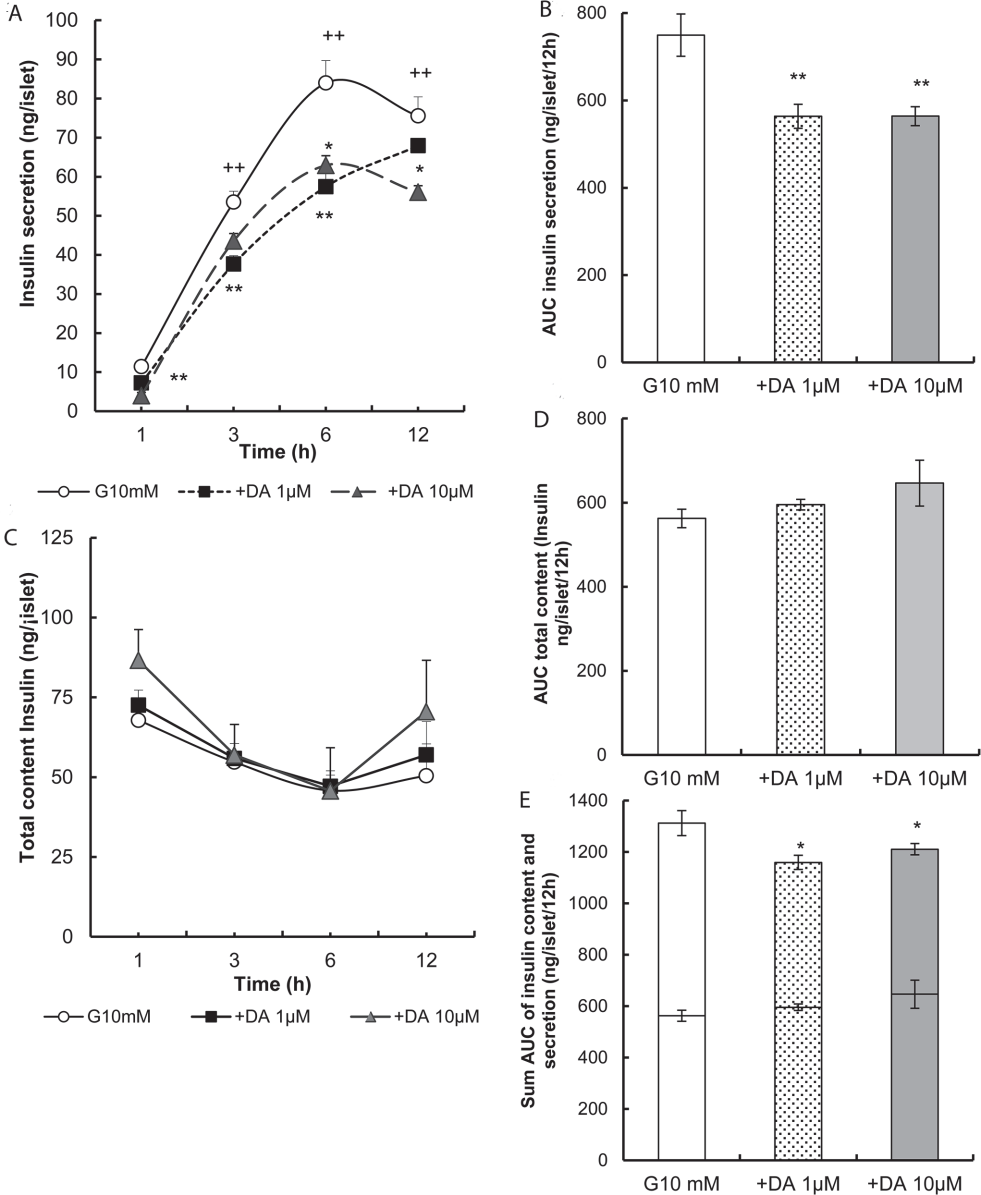
Effects of dopamine on insulin content and release. **(A)** Inhibition of insulin secretion in overnight cultured rat islets followed by treatment in glucose with dopamine 1 μM and 10 μM contrasted with glucose 10 mM as a control. Insulin secretion was then monitored at 1, 3, 6 and 12 h. **(B)** Absolute values AUC of insulin secretion were calculated for 10 mM glucose, 1μM and 10μM dopamine. **(C)** The insulin content of the islets used to study insulin secretion was determined at the end of the culture at 1, 3, 6 and 12h. **(D)** Absolute values of AUC of insulin content were calculated for 10 mM glucose and 1μM and 10μM dopamine. **(E)** This shows the sum AUC of insulin content (inferior portion) and insulin secretion (superior portion) by the same islets during treatment with dopamine (1 and 10 μM) *versus* glucose 10 mM. Absolute values are represented as means ± SEM for 15 batches of islets (10 islets per batch) from 2 experiments (*p<0.05 and **p<0.01).

Fig 1A shows the insulin levels in the culture medium from 1 h to 12 h following incubation with 10mM glucose. The insulin-levels in the medium ranged from 11.4±0.8 to 83.9±5.8 ng/islet, with a significant increase (p<0.01) at 3, 6 and 12 h in comparison with 1 h. The area under the curve (AUC) (Fig 1B) under the same conditions was 749.4± 48.3 ng/islet/12h. Treatment with dopamine induced a reduction in insulin release to the medium at all times and for all doses assayed (at 1, 3 and 6 h p<0.01 and at 12 h p<0.05 *versus* 10 mM glucose). The AUC of insulin secretion with time was decreased by about 25% for both doses of dopamine (p<0.01 n = 4, Fig 1B).

### Total content of insulin

The insulin contents of the islets used to study insulin secretion and determined at the end of the culture at 1, 3, 6 and 12 h, either alone or in the presence of dopamine, are shown in Fig 1C. In these conditions, dopamine did not change the total content of insulin significantly in comparison with the control. These data were corroborated when the AUC was determined (Fig 1D). However, the sum of the AUCs of total contents and insulin release exhibited significant differences after treatment with dopamine (p<0.05, Fig 1E).

### Insulin-positive beta-cells in isolated islets

**Fig 2:**
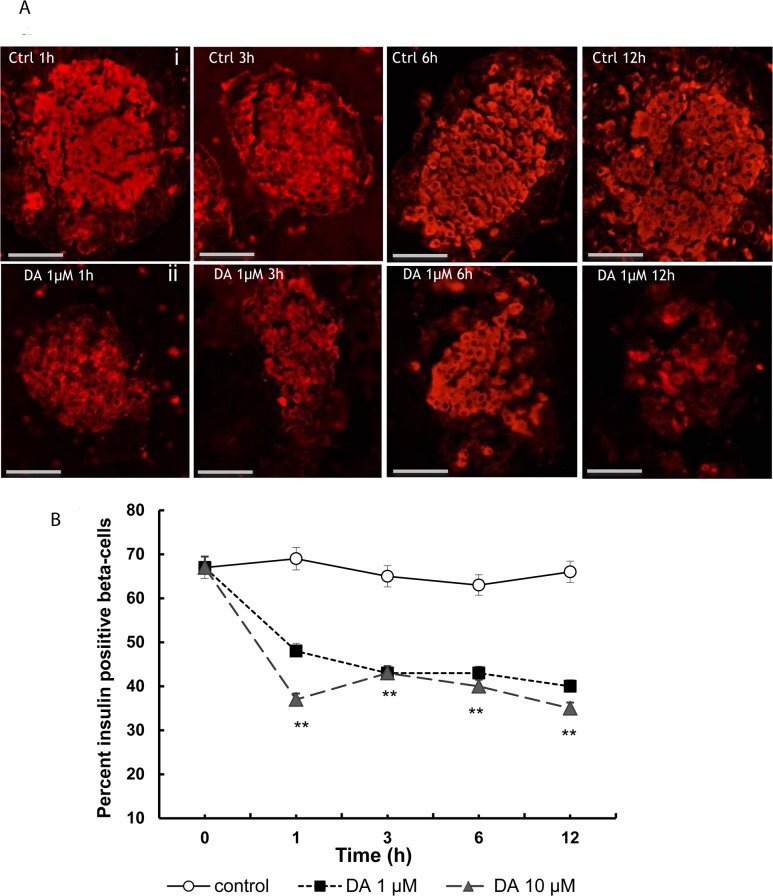
Inmunocytochemical analysis of insulin-positive cells in isolated islets treated with dopamine. **(A)** Micrographs showing some immunocytochemical staining patterns for insulin (red) in control islets (i), and dopamine-treated islets at 1, 3, 6 and 12 h (ii). **(B)** Plot showing the decrease induced by dopamine in the percentage of insulin-positive cells at the different time-points assayed; from 1 to 12 hours of treatment a significant decrease (*p<0.05, **p<0.01 with respect to their respective controls) was observed. Scale bar: 50 μm.

Insulin-positive cells were found mainly in the core of the islets (Fig 2A-i). Treatment with dopamine modified the intensity of the immunocytochemical reaction and the number and size of positive cells (Fig 2A-ii). In the control islets, the percentage of insulin-positive cells (Fig 2B) was very similar for all time-points assayed, ranging from 67± 2.5 to 66 ±2.4). In the islets treated with dopamine, the percentages of insulin-positive cells were decreased for all doses and time-points assayed (p<0.01 at 1, 3, 6 at 12 hours *versus* controls).

**Fig 3:**
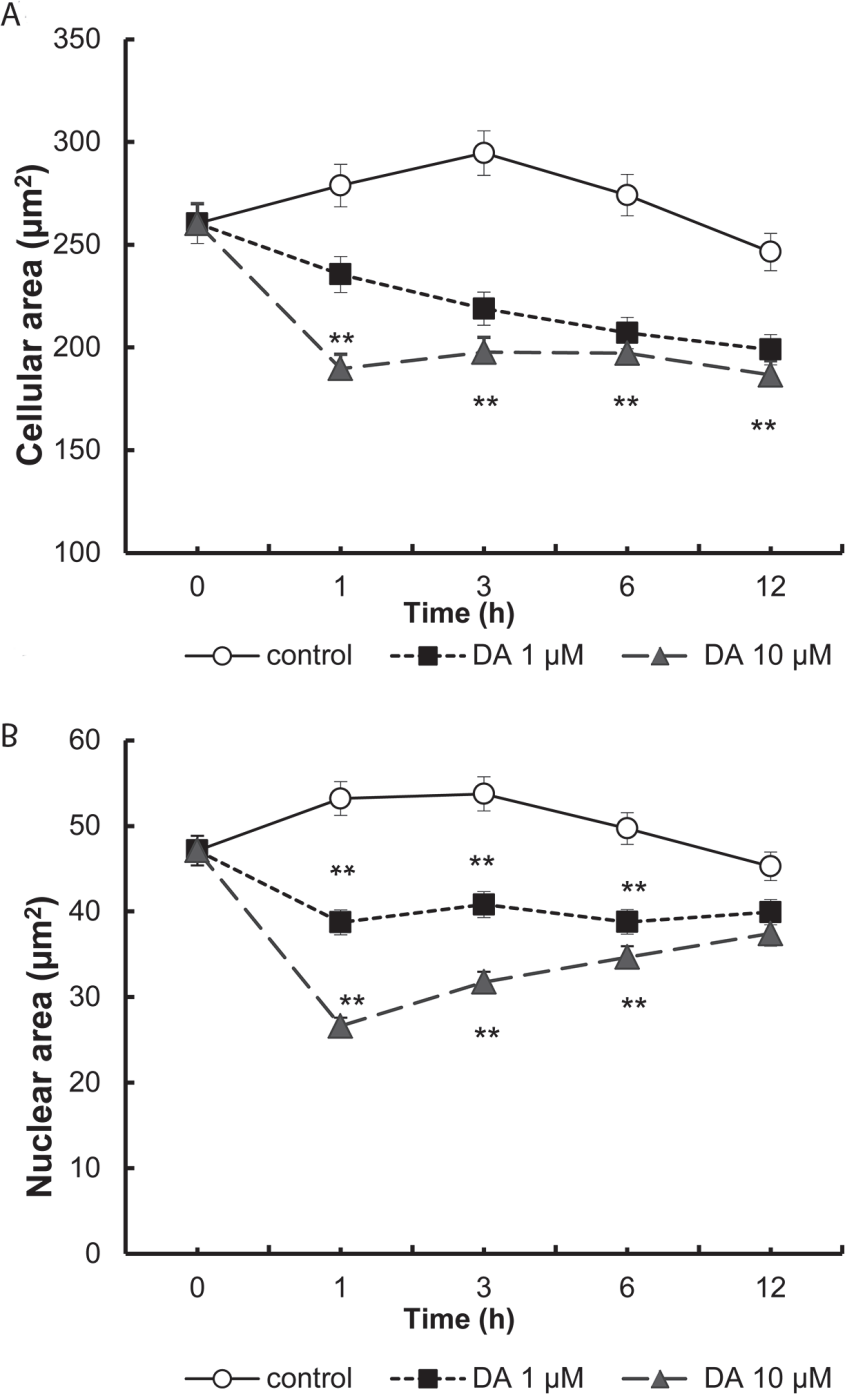
Morphometric effect induced by treatment with dopamine on cellular (A) and nuclear (B) area of beta cells. Histological sections were selected from micrographs of cross sections of each islets (see methods). In each section the cellular and the nuclear cell profiles from insulin-positive cells were plotted, allowing the surface of the cell and nuclear areas to be calculated after calibration of the Image J application. Cellular and nuclear area from10 islets per study group, and 100cells per islet were measured in 10 mM glucose, 1μM and 10 μM dopamine. (area: μm^2^, **p<0.01).

In the islets, the presence of dopamine decreased the cellular (Fig 3A) and nuclear areas of insulin-positive cells significantly for all doses and time-points assayed (Fig 3B), with a reduction of approximately 30% in the cellular area (**p<0.01) and of 45% in the nuclear area.

### Cellular proliferation rate of insulin-positive beta cells in isolated islets

**Fig 4:**
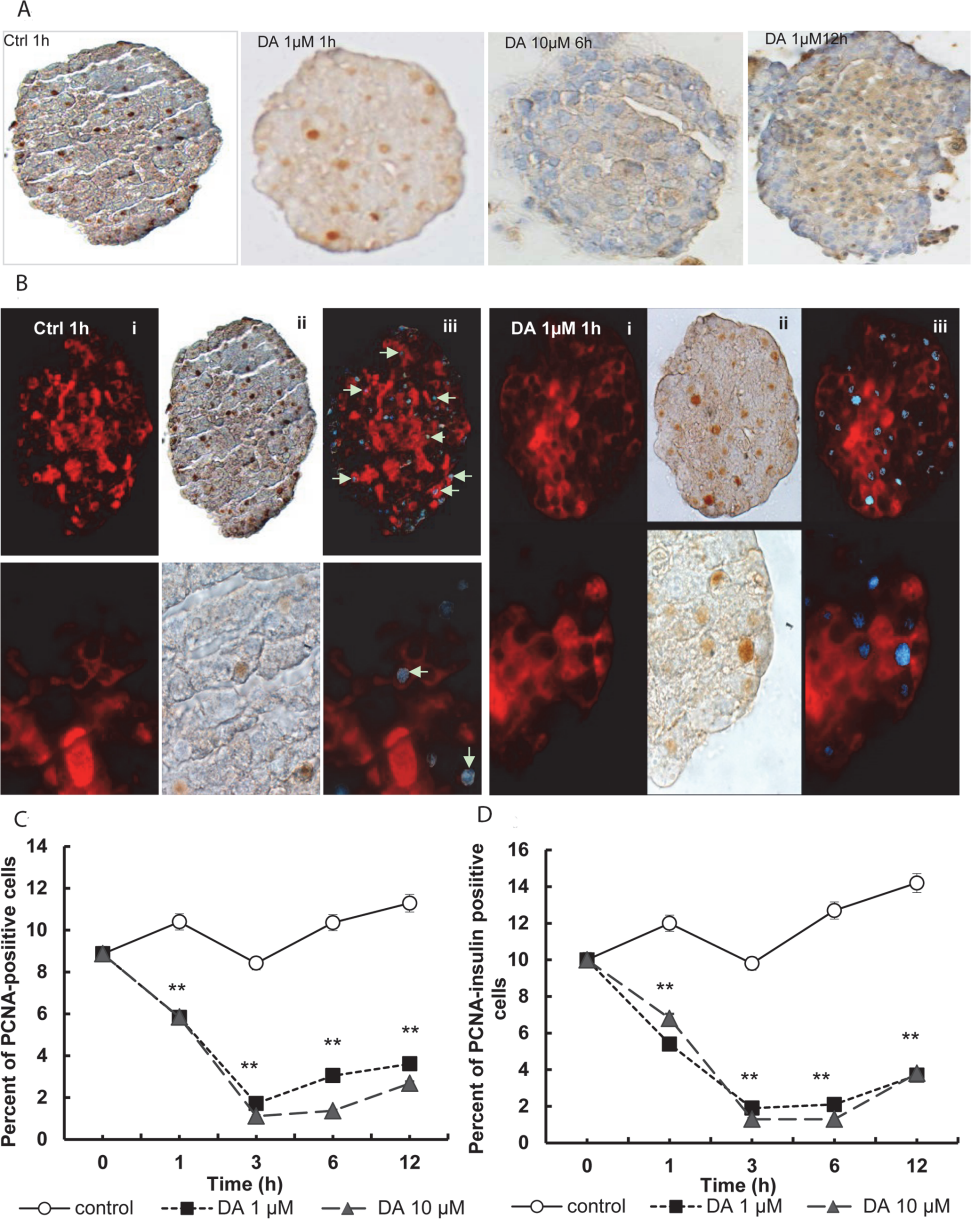
Effect of dopamine on cellular proliferation rate of insulin-positive beta cells in isolated islets. **(A)** After double immunocytochemical staining, the nuclei of PCNA-positive cells appear brown (ii). **(B)** The proliferating insulin-positive cells (i, red) show brown-stained nuclei (ii), and the count of positive cells and calculation of the percentages of insulin-positive cells in proliferation was enabled after combining both images following digital transformation of the brown colour to blue colour (iii). 4000 cells per group were counted to obtain the statistical values. **(C)** Percentage of PCNA-positive cells out of the total number of cells (alpha, beta and delta cells), and **(D)** percentage of PCNA- and insulin-positive cells out of the total number of insulin-positive cells in the control pancreatic islets and those treated with 1μM and 10 μM dopamine **p<0.01.

The micrographs in Fig 4 show the PCNA-positive cells (brown-stained nuclei) and insulin-positive cells (red-stained cells). In order to facilitate the visualization of proliferative insulin-positive cells, a combination of both images was carried out following digital transformation (adobe Photoshop CS2) of the brown colour to blue (shown with arrows, Fig 4B-iii).

Treatment with dopamine significantly decreased the percentage of PCNA-positive cells at all time-points and doses studied (p<0.01 *vs*. glucose control). At 3 h of treatment the percentage was decreased by about 80% (Fig 4C) and this was very similar between the two doses tested.

When only insulin and PCNA-positive cells were considered, the proliferation rates of insulin-positive cells were decreased after treatment with dopamine (p<0.01 *vs*. control) for all doses and time-points assayed. At 6 hours, dopamine reduced proliferation at 90% as compared with the controls (p<0.01 *vs*. controls).

### Study of cellular apoptosis by double immunocytochemical labelling for insulin and active Caspase-3 in beta cells

**Fig 5:**
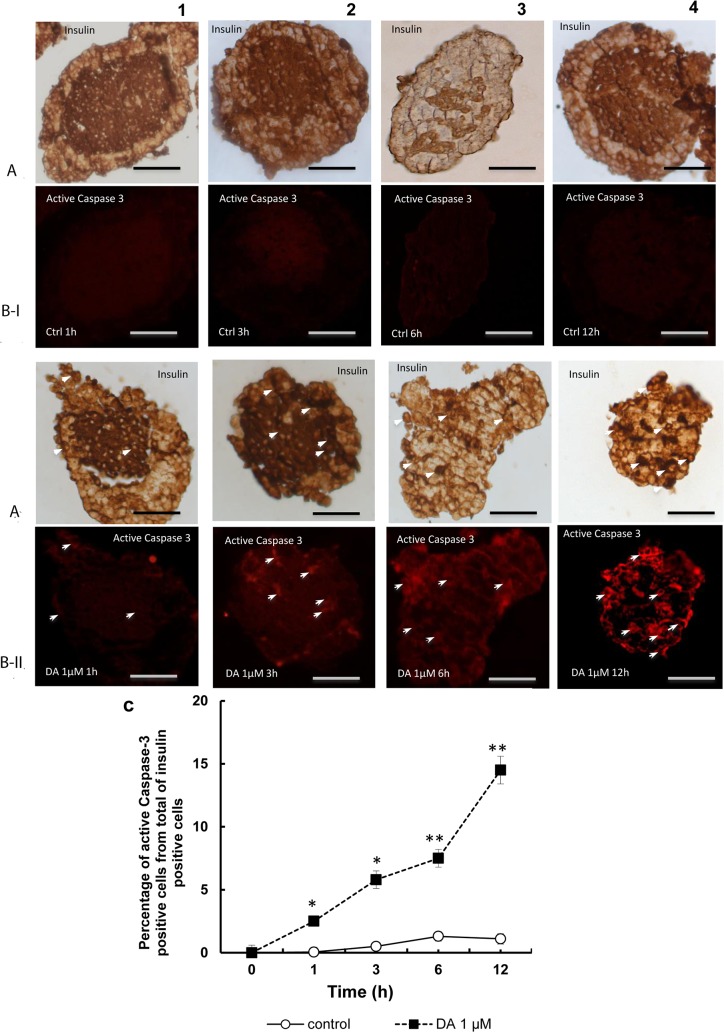
Apoptosis of beta cells treated with dopamine after labelling for active Caspase-3. Images obtained by double immunohistochemistry for insulin **(A)** (brown) and active caspase-3 **(B)** (fluorescence in red) in control islets (I), and dopamine-treated islets at 1, 3, 6 and 12 h (II). As can be observed in the micrographs there is minimal reaction in islets with glucose alone (BI-1, 2, 3, and 4). Treatment with dopamine induced positivity for caspase-3 (B-II 1, 2, 3 and 4), The intensity of the fluorescent signal was increased according to increase the time point of study. **(C)** Plot showing the increase induced by dopamine 1μM in the percentage active caspase-3 positive cells from total of insulin positive cells at the different time-points assayed. From 1 to 12 hours of treatment a significant increase (*p<0.05, **p<0.01 with respect to their respective controls) was observed. Scale bar: 50 μm.

Fig 5 shows images obtained with double immunohistochemistry for insulin (brown, Fig 5A) and active Caspase-3 (fluorescence in red, Fig 5B) where practically the absence of active Caspase-3 can be observed in control islets at all times (Fig 5B-I), together with the induction of the expression of active Caspase-3 after treatment with dopamine from 1 h up to 12 h (Fig 5B-II). In the control islets, the percentage of active Caspase-3 positive cells from total of insulin positive cells (Fig 5C) was imperceptible and similar for all time-points assayed. In the islets treated with dopamine, the percentages of active Caspase-3 positive cells from total of insulin positive cells were increased for all doses and time-points assayed (p< 0.05 at 1, 3 and p<0.01 at 6 at 12 hours *versus* controls).

## Discussion

Neurotransmitters, in particular catecholamines, play an important role in insulin secretion [26–28]. In recent years several advances have been made in the field and the components required for the synthesis, storage and secretion of dopamine in beta cells have been identified [15–18]. This has led to the proposal of a possible auto-paracrine regulation of insulin secretion by this neurotransmitter.

As could be expected, the results of this study show that exogenous administration of dopamine at pharmacological doses induces a significant and sustained decrease in the secretion rate of insulin in rat islets; this is accompanied by minimum changes in the total content of the hormone.

It has been reported that dopamine could induce stimulatory or inhibitory dose-dependent effects on insulin secretion [3]; however, our findings are in agreement with recent descriptions made in rodents and humans [12,13,19,29]. Moreover, this inhibitory response could be blocked with selective and nonselective dopamine antagonists [10, 12, 20]. Similar effects of ziprasidone and haloperidol (antagonists of dopamine receptors) on insulin secretion have been observed in our laboratory (data shown in S1 Fig).

The fact that dopaminergic receptors block the inhibitory action of dopamine on insulin release in pancreatic islets [12] supports the notion that the effect observed in the present study would be mediated through such receptors, whose presence in islets has been widely documented [13,14,20,22].

The inhibitory response along time could correspond to an increase in total insulin reserves if physiological balance has been preserved in the islets [24]. Here, on calculating the sum of the AUCs of the total contents and insulin secretion in the same islet we observed that it was significantly reduced after treatment with dopamine, suggesting that this latter could be involved in the regulation of the mass/and/or survival of beta cells.

The population of pancreatic beta cells is modified as a function of the physiological conditions, for which a balance must be maintained between cell proliferation and apoptosis [30], and adaptive changes may arise in order to conserve normoglycemia [31]. Our immunocytochemical study is the first reporting that the number of insulin-positive cells was reduced to a considerable extent following treatment with dopamine in comparison with the controls, suggesting that dopamine could be involved in the maintenance of these pancreatic cells.

A decline in the population of beta cells could help to explain the present findings concerning insulin, such as the decrease in the release of the hormone to the culture medium and the decrease in the total AUC in islets. This decrease in the number of cells was accompanied by a decrease in the cellular and nuclear areas of insulin-positive cells, suggesting that the activity of the cells decreases after dopamine treatment [32].

The signals required to facilitate the proliferation of beta cells involve specific combinations of well characterized molecules and/or transcription factors [33]. However, proliferative activity in routes such as the catecholaminergic pathway has been little studied. A recent work addressing pancreatic beta cells has shown that non-neural tyrosine hydroxylase is necessary for their development [34].

In ob/ob mice, the hyperplasia and hyperproliferative activity of islet beta cells clearly increases, partly as an adaptive response to the mechanisms that trigger insulin resistance [35,36]. The use of dopaminergic agonists in this model attenuated proliferative activity to a significant extent and normalized the hyperglycemia and FFA levels [35].

When we observed the proliferative activity of islets by means of immunocytochemical double labelling, after dopamine treatment we noted an important decline in the proliferation of PCNA-positive cells, both in the total cell number and in insulin-positive cells. These observations are consistent with previous reports in the literature. Despite this, in KO Drd2^-/-^ mice a reduction in the mass of beta cells and a decrease in their proliferative activity measured as PCNA have been observed [20], although such effects disappeared with age. Additionally, these results are difficult to compare with our own *in vitro* findings since the Drd2^-/-^ KO animals displayed chronic hyperprolactinemia, whose main pancreatic effect is to increase the proliferation of beta cells [37].

Activation of the proteolytic caspase cascade is a clear sign of cell apoptosis [38]. In beta cells, this is tightly regulated and increases slowly but steadily [39]. Moreover, it is known that both hypo- and hyperglycemia give rise to a loss of beta cell differentiation, alter stimulus-secretion coupling and increase the apoptotic rate of these cells [40–42]. These events contribute to the pathogenesis of type 2 diabetes mellitus [43, 44]. *In vitro*, the function and survival of rodent beta cells is conserved optimally in culture in the presence of intermediate (2–10mM) glucose concentrations [40] and acute and subacute stimulation of glucose in these conditions has beneficial effects on their phenotype [40,42,45]. With a view to preserving cell viability, we used 10mmol/l glucose as a control in our study.

Little is known about the possible involvement of dopaminergic signals in apoptosis in pancreatic islets. Different lines of investigation have demonstrated that catecholamines, including dopamine, are able to induce apoptosis in other types of tissue [46, 47].

This study is the first analysing the role of dopamine in the apoptosis of beta cells and observed that an increase in apoptosis, measured as the expression of active caspase-3, occurred in the presence of the neurotransmitter. These findings resemble those reported previously in other cell models [23, 48].

Recently, several studies have provided evidence of a negative feedback in dopaminergic signalling. Thus, in beta cells dopamine would be synthesized from circulating L-dopa and could be secreted together with insulin, serving as an auto-paracrine signal, and could induce the inhibition of glucose-stimulated insulin secretion [14,49]. *In vitro* experiments on human islets also suggest a new auto-paracrine regulatory circuit mediated by dopamine for insulin secretion [12].

## Conclusions

These results highlight the complex endocrine actions of the dopamine in the pancreas. The present results show that dopamine decreases insulin release, increases apoptosis, and decreases proliferation in isolated islets, suggesting that at least at pharmacological doses it could negatively modulate the maintenance of beta pancreatic cells, inhibiting cell proliferation and stimulating apoptosis. Moreover, dopaminergic dysregulation could eventually participate in the development of glucose intolerance. This is could be proposed to play a role in islet maintenance and survival and hence in the synthesis and secretion of the hormone.

## Supporting Information

S1 Fig Effects of antagonists of dopamine on insulin release and content. **(A)** Role of haloperidol 5μM on insulin secretion and in the presence of dopamine compared with inhibition of insulin secretion induced by dopamine 1μM. **(B)** Role of ziprasidone 10μM on insulin secretion and in the presence of dopamine compared with inhibition of insulin secretion induced by dopamine 1μM. All solutions contained 10 mM glucose that was used as a control. Insulin secretion was then monitored at 1, 3, and 6 h**. (C)** Absolute values AUC of insulin secretion were calculated for 10 mM glucose, 1μM dopamine, 5μM haloperidol, dopamine+haloperidol and **(D)** 10μM ziprasidone, and dopamine+ziprasidone. Absolute values are represented as means ± SEM for 14 batches of islets (10 islets per batch) from 2 experiments (*p<0.05 and **p<0.01).
(TIF)
