# Supplementary figures and images for: Dopamine Modulates Insulin Release and Is Involved in the Survival of Rat Pancreatic Beta Cells
Source: PLoS One. 2015 Apr 17;10(4):e0123197. doi: 10.1371/journal.pone.0123197 (PMC4401745; doi:10.1371/journal.pone.0123197)

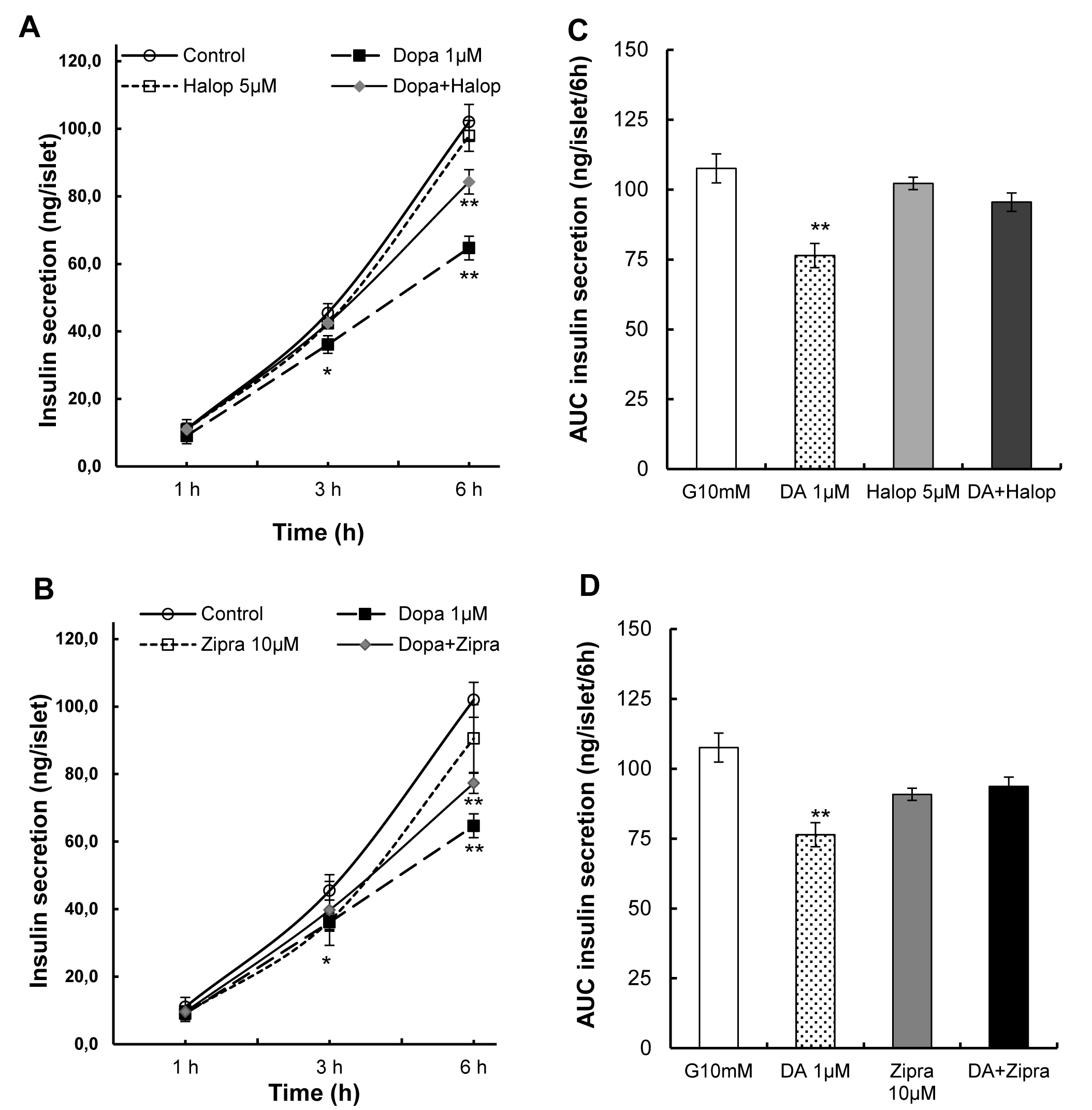

Supplement: S1 Fig — (A) Role of haloperidol 5μM on insulin secretion and in the presence of dopamine compared with inhibition of insulin secretion induced by dopamine 1μM. (B) Role of ziprasidone 10μM on insulin secretion and in the presence of dopamine compared with inhibition of insulin secretion induced by dopamine 1μM. All solutions contained 10 mM glucose that was used as a control. Insulin secretion was then monitored at 1, 3, and 6 h. (C) Absolute values AUC of insulin secretion were calculated for 10 mM glucose, 1μM dopamine, 5μM haloperidol, dopamine+haloperidol and (D) 10μM ziprasidone, and dopamine+ziprasidone. Absolute values are represented as means ± SEM for 14 batches of islets (10 islets per batch) from 2 experiments (*p<0.05 and **p<0.01). (TIF) [file pone.0123197.s001.tif]
